# Acute Kidney Injury Secondary to Rhabdomyolysis and COVID-19: A Case Report and Literature Review

**DOI:** 10.1155/2021/5528461

**Published:** 2021-08-02

**Authors:** Vishaka K. Chetram, Akram I. Ahmad, Saira Farid, Tanuj Sood

**Affiliations:** Department of Internal Medicine, Washington Hospital Center, Washington, DC 20010, USA

## Abstract

The viral infection causing COVID-19 most notably affects the respiratory system but can result in extrapulmonary clinical manifestations as well. Rhabdomyolysis-associated acute kidney injury (AKI) in the setting of COVID-19 is an uncommon complication of the infection. There is significant interest in this viral infection given its global spread, ease of transmission, and varied clinical manifestations and outcomes. This case report and literature review describes the symptoms, laboratory findings, and clinical course of a patient who developed AKI secondary to rhabdomyolysis and COVID-19, which will help clinicians recognize and treat this condition.

## 1. Introduction

The novel coronavirus which emerged in late 2019, designated as severe acute respiratory syndrome coronavirus 2 (SARS-CoV-2) and responsible for COVID-19, has infected more than 85 million people worldwide and has grown into a global pandemic. The incubation period, disease severity, initial presentation, and laboratory findings are varied [1–4]. Multiorgan complications include respiratory failure, cardiac arrhythmias, cardiomyopathy, encephalopathy, thromboembolic events, and acute kidney injury (AKI) [5–7].

Kidney involvement in the setting of COVID-19 manifests as hematuria, proteinuria, and/or an AKI, which is associated with increased mortality and more severe infections [8, 9]. Estimates of the incidence of AKI range from 17% to 37% and between 5–15% require kidney replacement therapy [8, 10]. The etiology of kidney dysfunction remains unexplored and may be the result of direct viral infection, cytokine release, altered hemodynamics, or multifactorial, including rhabdomyolysis. Characterized as a clinical syndrome of muscle weakness, myalgia, tea-coloured urine, and muscle swelling, rhabdomyolysis is defined with a creatine kinase value greater than 1000 IU/L or higher than 5 times the upper limit of normal [11]. Rhabdomyolysis occurs due to trauma, drugs, electrolyte abnormalities, and also rarely, viral infections such as influenza A and B, coxsackievirus, Epstein–Barr, herpes simplex, parainfluenza, adenovirus, echovirus, HIV, cytomegalovirus, and most recently, COVID-19 [12, 13]. Thus, our aim in this case report is to present the symptoms, laboratory findings, and clinical course of a patient who presented with AKI secondary to rhabdomyolysis and COVID-19 and provide a literature review of the reported cases.

## 2. Case Report

A 62 year-old-African American male patient presented to the emergency department with a 3-day history of general feeling of malaise, poor appetite, decreased urine output, and blood in his urine. His comorbidities include hypertension, morbid obesity (BMI of 39.6), and suboptimally controlled type II diabetes. He was socially distancing alone at home and reported no known sick contacts. He denied taking any anticoagulation medication and had no symptoms of urinary urgency or dysuria. He also denied recent strenuous activity, alcohol ingestion, or taking any over-the-counter supplements. Home medications included aspirin 81 mg, glargine 25 units daily, hydrochlorothiazide-lisinopril 12.5mg–20 mg daily, pioglitazone 30 mg, and simvastatin 20 mg daily.

**Figure 1:**
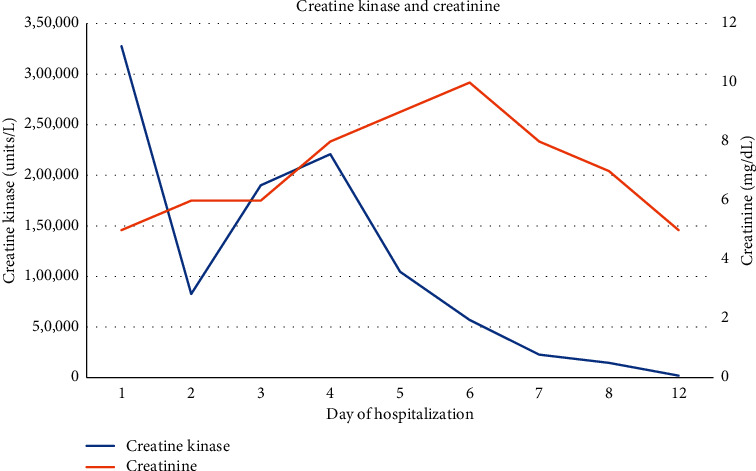
Trend of creatinine kinase and creatinine.

On presentation, he was febrile to 38.1°C with a heart rate of 103, respiratory rate of 20, blood pressure of 91/58 mmHg, and saturating well on room air. Physical examination yielded a patient with a large body habitus and mild respiratory distress. Pertinent initial laboratory findings revealed a sodium of 132 mmol/L, potassium 4.5 mmol/L, magnesium 2.0 mg/dL, phosphorus 8.9 mg/dL, corrected calcium 4.4 mg/dL, BUN 54 mg/dL, creatinine 4.90 mg/dL (baseline of 1.1 mg/dL), glucose 372 mg/dL, aspartate aminotransferase (AST) 1077 units/L (baseline of 16 units/L), alanine aminotransferase (ALT) of 158 units/L (baseline of 24 units/L), alkaline phosphatase 45 units/L, total bilirubin 0.4 mg/dL, glomerular filtration rate (GFR) 14 mL/min, and lactic acid of 2.1 mmol/L. Urine albumin-to-creatinine ratio one month prior to presentation was 30 mg/g. Creatinine kinase was 327, 629 units/L, and peaked on presentation. Hemoglobin A1C glycosylated was 12.9%. D-dimer was 2.01 mcg/mL FEU. Arterial blood gas showed a pH of 7.35, CO2 of 31 mmHg, and bicarbonate of 17.1 mmol/L. C-reactive protein (CRP) was 136.39 mg/L, and thyroid-stimulating hormone (TSH) was 1.397 uIU/mL. Hepatitis viral panel was obtained and negative. Urine toxicology was negative. Urine analysis showed a moderate amount of blood and 19/hpf red blood cells. The progression of creatinine and creatinine kinase is depicted in Figure 1, respectively.

**Figure 2:**
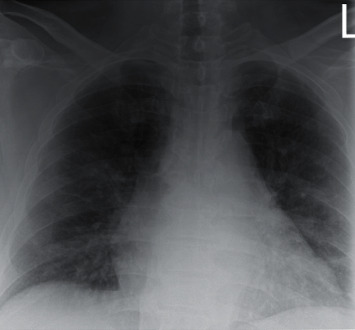
Chest X-ray showing multifocal patchy airspace disease, COVID-19.

Point-of-care ultrasound of the bladder showed <100 mL of urine. Ultrasound of the abdomen showed hepatomegaly with fatty infiltration and no acute cholecystitis or choledocholithiasis, as well as no hydronephrosis. The initial chest radiograph was significant for multifocal patchy airspace disease suggestive of atypical pneumonia or viral infection including COVID-19 (Figure 2).

Initial COVID-19 RNA PCR was negative. Respiratory viral panel for influenza and respiratory syncytial virus (RSV) was also negative. He was given a bolus of 2 L lactated ringer (LR) and started on maintenance LR at 200 mL/hour as well as sevelamer 2400 mg three times a day. For the first 48 hours, he was anuric. He also received ceftriaxone and azithromycin for community-acquired pneumonia coverage. His anion gap and metabolic acidosis resolved shortly after administration of IV fluids. Following the first 48 hours, his urine output increased to 600 mL over 24 hours on the third day and was red tinged. Urine output decreased to 210 mL and 150 mL per 24 hours on hospitalization days four and five, respectively. On the third day of his hospitalization, given the elevated D-dimer and concerning chest radiograph findings, COVID-19 PCR was repeated and found to be positive.

Despite his positive test, the patient did not have respiratory symptoms and continued to oxygenate well on room air. On day 3, continuous renal replacement therapy (CRRT) was initiated due to persistent low urine output, worsening kidney indices, and volume overload. He received 3 consecutive days of CRRT while maintenance fluids were continued at 100 mL per hour. His urine output gradually increased, and CRRT was interrupted. By day 5, his creatine kinase downtrended to 57, 074 units/L with improved serum creatinine to 5.46 mg/dL. At the end of the first week of his hospitalization, his urine appeared clear and total 24-hour output was 700 mL. Given his improvement in urine output and downward trend in creatinine, CRRT was stopped.

**Figure 3:**
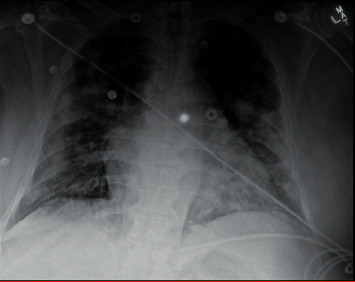
Chest X-ray on day 14 showing worsening bilateral infiltrates.

On day 8 of his hospitalization, his oxygen levels desaturated to 87% while walking to the bathroom and he was placed on 2 L supplemental oxygen via a nasal cannula. Maintenance fluids were also discontinued at this time, and he developed a fever to 38.1°C. Chest X-ray showed worsening bilateral infiltrates (Figure 3).

At this point in his clinical course, his blood pressures remained elevated, ranging from 170/80 to 200/90, and antihypertensive medication doses were progressively increased up to amlodipine 10 mg daily and hydralazine 100 mg thrice a day. On day 12, the patient went into acute hypoxic respiratory failure, developed tachycardia with a heart rate of 120s beats per minute and tachypnea with a respiratory rate of 30. An arterial blood gas (ABG) was obtained which showed a pH of 7.41, CO_2_ 29 mmHg, bicarbonate 18.4 mmol/L, and PaO_2_ of 82 mmHg. He was placed on BiPAP at 60% FiO_2_, broad-spectrum antibiotics for pneumonia coverage, and heparin drip for suspected pulmonary embolism and was transferred to the intensive care unit (ICU).

In the ICU, he was started on a 10-day course of dexamethasone and weaned to a high-flow nasal cannula after 2 days. Remdesevir was never given due to kidney dysfunction. He was also intermittently treated with diuretics. By day 14 of his hospitalization, his daily urine output was 2.9 L and clear in color. He received an echocardiogram which showed an ejection fraction of 65–70% with no valvular or diastolic dysfunction. Ultrasound of the lower extremities was negative for deep vein thrombosis. As his respiratory status improved, he was weaned to 6 L supplemental oxygen via a nasal cannula and was transferred out of the ICU. The patient gradually improved as he completed the 10-day course of dexamethasone, and at the time of discharge, 26 days after his presentation, his creatinine gradually decreased to 1.16 mg/dL and he was sent home, on room air.

## 3. Discussion

COVID-19-related kidney dysfunction occurs in a number of ways [14] and can manifest as (1) prerenal AKI from volume depletion or cardiorenal syndrome, (2) acute tubular injury in the setting of circulatory collapse, (3) thrombotic microangiopathy secondary to hypercoagulation, (4) collapsing glomerulopathy in APOL1 gene variants, and (5) myoglobin cast nephropathy due to rhabdomyolysis. Emerging literature has connected COVID-19 to the development of rhabdomyolysis and acute kidney injury, but only few cases have been reported (Table 1) and the physiology link remains unknown.

The proposed hypotheses include direct invasion of muscle tissue, release of toxic cytokines, namely, TNF-alpha, and destruction of muscle cell membranes by circulating toxins [25]. Approximately 5% of hospitalized COVID-19 patients develop an AKI [26], and up to 40% of those are admitted to the ICU [27]. Studies have indicated that the development of stage II or III AKI incurs a poor prognosis with mortality rates as high as 70% in those requiring kidney replacement therapy [26, 28, 29]. The patient in this case report developed stage III AKI secondary to his rhabdomyolysis which was the presenting symptom of COVID-19. Although he required kidney replacement therapy, he fortunately obtained complete recovery despite a prolonged hospital course and also requiring ICU level of care.

Rhabdomyolysis has been described as a late complication of COVID-19 [30], but in our case, it was the presenting clinical manifestation even before respiratory symptoms developed and in light of a negative initial COVID-19 test. Of the reported cases, the classic triad of weakness, myalgia, and tea-coloured urine was not seen in any patients and only 7 of 16 reported one symptom, most commonly myalgia. In this case report, the patient presented with tea-coloured urine, decreased urine output, and general malaise and weakness. Myalgia is a common complaint of viral infections, including influenza. Thus, in patients presenting with myalgia and respiratory symptoms or high suspicion of COVID-19, we recommend evaluating serum creatine kinase and serum creatinine and closely monitoring urine output. The development of rhabdomyolysis and AKI does not appear to have a predilection in patients with certain comorbidities or age. Additionally, it appears that peak creatinine kinase is not associated with in-hospital mortality. In this case, peak creatine kinase was 327, 629 units/L, the second highest of the reported cases, and the patient recovered completely. This contrasts with the cases reported by Singh et al. where peak creatinine kinase was less than <10,000 units/L and patients died during their hospitalization.

The favourable outcome of our patient may be linked to early identification and IV fluid therapy.

Regarding management of rhabdomyolysis, the mainstay continues to be correction of intravascular volume depletion and prevention of intratubular cast formation with fluid resuscitation. The initial recommended rate is 1 L/hour followed by 500 mL/hour thereafter with either isotonic saline or lactate ringers, although there is no strong evidence supporting either of these IV fluid formulations [31]. The goal of therapy is increased urine output; however, clinicians should be cognizant of volume overload in the setting of oliguria [32]. Additionally, electrolyte abnormalities such as hyperkalemia, hyperphosphatemia, and hypercalcemia or hypocalcaemia should be monitored closely and addressed quickly. Further evidence is needed on target therapies to prevent rhabdomyolysis-associated AKI [33].

A potential limitation to this report is that the patient was taking 20 mg simvastatin as a home medication, a notable cause of myopathy and rhabdomyolysis. Statin-induced rhabdomyolysis is rare and occurs in <0.1% of cases [34]. Furthermore, in larger, follow-up studies, rhabdomyolysis-associated acute kidney failure only developed when statins were used in combination with cyclosporine, gemfibrozil, protease inhibitors, niacin, digoxin, and some antimicrobials [35], none of which this patient was taking at the time. Lastly, although myopathy can occur at any time during treatment with a statin, the onset of symptoms is usually within months of initiation of therapy. Approximately 2/3 of patients experience myopathy in the first six months of starting therapy [36], and the patient in this case report had been on statin therapy for eighteen months without changes in dose. Pioglitazone has been associated with rhabdomyolysis [37], although it appears to be at higher doses (75 mg/day) and rare. Risk factors for thiazolidinedione-induced rhabdomyolysis include concomitant therapy with fibrate, alcohol abuse, and asymptomatic mild creatinine phosphokinase elevation prior to initiating therapy. The patient in this report was on a lower dose of pioglitazone (30 mg/day) and had no further additional risk factors. Other causes of rhabdomyolysis were excluded with thyroid function tests and urine toxicology.

In summary, this is a unique presentation and complication of COVID-19 in a patient who initially tested negative for the virus. This case was unique in that the patient had a favourable prognosis and although being being admitted to the ICU, recovered completely. Most described cases presented with respiratory symptoms or were diagnosed with COVID-19 and were subsequently found to have rhabdomyolysis and kidney dysfunction, unlike the case in this report. This case report also details the clinical presentation, hospital course, and treatment which adds to the existing literature on the phenomenon. It is imperative for clinicians to be aware of the potential for COVID-19 patients to develop rhabdomyolysis, initiate early treatment, and minimize the kidney dysfunction.

## Figures and Tables

**Table 1:** Summary of the reported cases.

	Demographics	Clinical Presentation	Comorbidities	Pertinent labs	Clinical course and outcome
Taxbro et al. [15]	38-year-old male	1 week of fever, myalgia, nausea, emesis, dry cough, dyspnea, and abdominal pain	Type 2 diabetes Gout	CRP: 145 mg/dL Creatinine: 51 mmol/L D-dimer: 0.19 mg/dL No reported CK	Complete recovery after ICU admission requiring intubation LOS: 23 days
Valente-Acosta et al. [16]	71-year-old male	1 week of dry coughing, mild dyspnoea and fever, and myalgia and arthralgia, predominantly in his legs	Benign prostatic hyperplasia	CRP: 2.9 mg/dL Creatinine: 1.68 mg/dL LDH: 541 U/L D-dimer: 983 ng/mL CK peak: 8,720 U/L	Complete recovery after ICU admission requiring intubation LOS: 16 days
Singh et al. [17]	34-year-old male	Fever, cough, dyspnea, and weakness	Prediabetes	— Creatinine 0.89 mg\dl — — CK peak: 5,454 U/L	Died in hospital LOS: not reported
	71-year-old male	Fever, cough, and dyspnea	Hypertension Schizophrenia Seizures	— Creatinine: 4.1 mg/dL — — CK peak: 10,247 U/L	Died in hospital LOS: not reported
	88-year-old male	Confusion	Diabetes Hypertension	— Creatinine: 2.25 mg/dL — — CK peak: 2,628 U/L	Died in hospital LOS: not reported
	36-year-old male	Fever, cough, and dyspnea	None	— Creatinine: 1.03 mg/dL — — CK peak: 5,531 U\L	Died in hospital LOS: not reported
	39-year-old male	Myalgias, fever, cough, and dyspnea	Hypertension	— Creatinine: 3.8 mg/dL — — CK peak: 4,330 U/L	Died in hospital LOS: not reported

Husain et al. [18]	38-year-old male	Sever, cough, dyspnea, and myalgia	Not reported	CRP: 98.3 mg/dL Creatinine: 1.5 mg/dL LDH: 398 U/L — CK peak: 33,000 U/L	Complete recovery after ICU admission LOS: 3 months
Chedid et al. [19]	51-year-old male	2 days of diffuse myalgias, dry cough, and mild chills	Hypertension Type 2 diabetes OSA CKD II	CRP: 98.3 mg/dL Creatinine: 2.48 mg/dL LDH: 2150 U/L — CK peak: 464,000 U/L	Dialysis as outpatient LOS: not reported
Singh et al. [20]	67-year-old male	Fever and dyspnea	Hypertension	— Creatinine: 1.16 mg/dL LDH: 459 U/L D-dimer: 0.62 ng/mL CK peak: 19,773 U/L	Died in hospital LOS: 21 days
	39-year-old male	Fever, myalgia, dyspnea, and altered mental status	Hypertension	CRP: 98.3 mg/dL Creatinine: 3.8 mg/dL LDH: 907 U/L D-dimer: 1.92 ng/mL CK: 4,330 U/L	Died in hospital LOS: 1 day

Alejandro et al. [21]	89-year-old male	Dyspnea, fever, cough, and malaise	Hypertension, coronary artery disease, and heart failure with preserved ejection fraction	Creatinine: 2.0 mg/dL LDH: 805 U/L — CK: 2,751 U/L	Complete recovery LOS: 12 days
Pellegrini et al. [22]	34-year-old male	2 days of emesis, sore throat, nonproductive cough, and dyspnea	Not reported	CRP: 3.3 mg/dL Creatinine: 1.4 mg/dL LDH: 854 U/L — CK: 73,922 U/L	Complete recovery LOS: 8 days
Chong and Saha [23]	37-year-old male	2 days of dyspnea and fatigue	Not reported	— Creatinine: 5.0 mg/dL — — CK peak: 35,000 U/L	Died in hospital LOS: 12 day
Chan et al. [24]	75-year-old female	4 days of weakness and decrease in appetite	Coronary artery disease, hypertension, and GERD	— Creatinine: 1.2 mg/dL — — CK: 2,767 U/L	Complete recovery LOS: not reported
	71-year-old male	Generalized weakness and leg twitching	Hypertension, seizure, CKD, and overactive bladder	— Creatinine: 5.6 mg/dL — — CK: 1,859 U/L	Hospitalized as inpatient LOS: undetermined

Chetram et al., 2021 (this manuscript)	62-year-old male	3-day history of general feeling of malaise, poor appetite, decreased urine output, and blood in his urine	Hypertension, type II diabetes, obesity	CRP: 136.39 mg/L Creatinine: 4.9 mg/dL — D-dimer: 2.01 mcg/dL CK peak: 327,629 U/L	Complete recovery LOS: 26 days
